# Influential factors for COVID-19 related distancing in daily life: a distinct focus on ego-gram

**DOI:** 10.1186/s12889-022-13336-0

**Published:** 2022-05-10

**Authors:** Kyu-Min Kim, Hyun-Sill Rhee

**Affiliations:** 1 Department of Public Healthcare Sciences, Graduate School, Korea University, 145 Anam-ro, Seongbuk-gu, Seoul, South Korea; 2 BK21FOUR R&E Center for Learning Health Systems, Korea University, 145 Anam-ro, Seongbuk-gu, Seoul, South Korea; 3 School of Health Policy and Management, College of Public Health Science, Korea university, 145 Anam-ro, Seongbuk-gu, Seoul, South Korea

**Keywords:** COVID-19, Distancing in daily life, Ego-State

## Abstract

**Background:**

The coronavirus disease 2019 (COVID-19) has necessitated that individuals comply with personal quarantine rules in daily life. South Korea is implementing the concept of “distancing in daily life” to raise awareness on personal quarantine measures, which is communicated through various media channels and platforms. The continued rise in COVID-19 cases demands that all individuals strictly adhere to personal quarantine rules. It is worth paying particular attention to the college student group, which has the highest percentage of confirmed cases among all age groups in South Korea. This group understands and practices “distancing in daily life” but with drastic variations among individuals. Previous studies have reported that the level of adherence to social norms is different according to each ego stated, and media exposure level is reported as a major influencing factor. Therefore, this study examined the media exposure level to COVID-19 prevention rules and its effect on the observance of distancing in daily life; it also verified the moderating effect of ego-gram on the relationship between media exposure level and distancing in daily life.

**Methods:**

The participants comprised Korean university students (men = 143, women = 188, *N* = 331) aged 18–30 years, who were recruited through an online survey. The survey was conducted over 20 days from January 27 to February 15, 2021. Data were analyzed using SAS (version 9.4) to calculate hierarchical regression.

**Results:**

First, media exposure level and distancing in daily life among Korean university students was above average. Second, media exposure level (β = .161, P < .01) was identified as the most influential factor for distancing in daily life. Third, ego-grams had a moderating effect (β = .136, P < .05) on the relationship between media exposure levels and distancing in daily life.

**Conclusions:**

This study examined the policy implications related to the development of diverse quarantine-related programs while considering influential factors and differences in how individuals’ compliance with quarantine rules were presented. Considering the situation in which new infectious diseases such as COVID-19 occur every 4–5 years, this study serves as a preparation for future pandemics and is an important framework to enhance the level of personal quarantine.

## Background

The coronavirus disease 2019 (COVID-19) was first reported in China in December 2019 and rapidly spread worldwide; about three months later, the World Health Organization (WHO) officially declared the outbreak of the virus a pandemic [1]. Since then, COVID-19 has significantly altered our daily life patterns, and has affected over 180 million people worldwide and caused more than 3.8 million deaths to date [2]. Owing to the rapid and high frequency of human-to-human transmission, the incidence and mortality of COVID-19 are still increasing rapidly worldwide [3]. The WHO has stated that, compared to other countries, South Korea has managed its cumulative confirmed cases and COVID-19 mortality rates relatively well. This is because of many quarantine policies such as the “K-Quarantine,” the ability to perform more than 3000 COVID-19 tests daily, and the establishment of a COVID-19 response system using ICT (Information and Communications Technology) [4, 5]. The positive outcome can be attributed to the implementation of preventative measures, but at its core, the voluntary participation of people is an important factor for success.

Therefore, this study measured and discussed the degree of actual involvement of individuals by focusing on “distancing in daily life” policies that require pure voluntary participation rather than mandatory compliance. Distancing in daily life involves adherence to personal-level quarantine rules by carrying out activities to block and prevent the spread of COVID-19 while ensuring that people’s daily lives and economic activities continue unencumbered [6]. Measures such as minimizing personal contact have been shown to reduce the spread of the virus [7–9], and are considered the most effective preventive method in situations where vaccines and therapeutics are not widely available [10, 11]. Distancing in daily life is a social norm that is defined as an “agreement on the range of common-sense behaviors allowed without legal restrictions,” which provide people with a set of rules about acceptable social behavior [6]. However, the influence of these rules may differ among groups [12–14], and there is a need to pay particular attention to the group comprising university students. These students are in the process of transitioning from immature adolescents to adults, and they must adapt to and overcome the changes in the social environment. Furthermore, peer groups are an important reference group and students are greatly influenced by social norms or behaviors shared by peers [15]. The distribution of COVID-19 confirmed cases by age shows that the 20–29 age group, which accounts for most college students, represents the highest percentage of confirmed cases among all age groups [6]. In short, the Korean university student group could be considered crucial in observing how people follow social norms, including quarantine rules.

With the rapid spread of COVID-19, various types of media have been reporting on related precautions on a daily basis. According to a previous study, the ratio of acquiring information through mass media was high among the various routes available to find specific information, such as COVID-19 prevention rules [16]. In addition, the search for and acceptance of information by individuals through social media in a crisis such as COVID-19 has a positive effect on behavioral change [17, 18]. Studies show that the media is very effective for not only understanding social norms but also for behavior correction, and the influence of media in our society is expected to increase as the level of media exposure increases [19–21].

Research on these stimuli have reported that emotional responses to external stimuli vary depending on individual personality characteristics [22]. In short, an individual’s “personality” represents a person’s unique way of behavior, which is formed and developed throughout their life; therefore, social solidarity is expected to improve if support is provided based on these characteristics. The ego-gram is mainly used as a method to measure an individual’s personality type. It is a personality theory proposed by Eric Burne based on Transaction Analysis and is also a communication evaluation scale developed by John Dusay through the application of the five human egos [18]. It is mainly used to identify individual behavioral characteristics and emotional problems, and to suggest improvement measures. Additionally, the evaluation takes as little as 5–10 minutes, thus resulting in high frequency of use [23, 24]. An ego-gram can express an individual’s psychological energy as a diagram, and personality types are defined in various forms. The characteristic that presents a large slope in the state of energy can lead to a personality imbalance; here, we must consider five bars that indicate the amount relevant to energy—CP (critical parent), NP (nurturing parent), A (adult), FC (free child), and AC (adapted child), which are all characterized by the individual’s ego. In short, CP refers to the ability to teach one’s beliefs or value judgments about oneself and others; NP refers to the ability to help nurture and protect others; A refers to the ability to accumulate information and judge things based on facts; FC refers to the ability to express one’s emotions freely; and AC refers to the ability to control one’s emotions and fit into society [25].

Major studies so far, especially in South Korea, have examined how the level of media utilization affects discriminatory attitudes toward the elderly [19], the effect of political participation based on social media exposure levels [20], the quality of life of university students by identifying their ego-grams [26], and the relationships between sociality, career planning, and volunteer activity satisfaction based on ego grams [22]. Another study [27] examined the relationship between a nurse’s ego state and interpersonal attitude, which showed that the A ego type was high and that interpersonal attitudes differed depending on the ego state. However, studies related to “distancing in daily life” are still insufficient. Research has been conducted in other countries on the influence of media attitudes on smoking cessation [28], the relationship between influenza prevention behavior and social norms [29], and the role and impact of the social media in the context of COVID-19 [30]. Additionally, the relationship between news, social distancing, and cognition in adults, which appeared to affect the level of trust in the news and caused a difference in the influence of social distancing, was analyzed [31]. Most studies dealt with individual causality, such as media exposure levels and attitudes (intention), while those dealing with media exposure levels, ego-grams, and attitudes (intention) in combination have been very poor. Particularly, few studies have linked COVID-19, distancing in daily life, media exposure levels, and ego-grams. Therefore, this study investigated the level of media exposure, distancing in daily life, and ego-grams of university students to analyze the relationship between media exposure level and distancing in daily life and how ego-grams can moderate this relationship. Understanding the personality characteristics of college students and the level of each factor is the basic data for measures to promote social norms such as distancing in daily life.

## Methods

### Study design

This descriptive study examined the media exposure level and degree of distancing in daily life among Korean university students and determined whether ego-grams could have a moderating effect on the relationship between these two variables.

### Study objectives

**Fig. 1:**
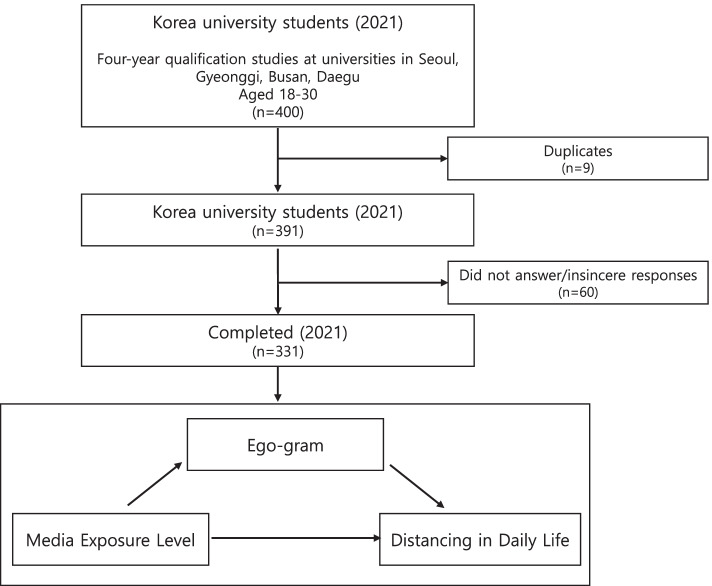
Survey Response Flowchart

In this study, an online survey was conducted among male and female college students aged 18 years or over and under 30 years, who were currently attending or taking a leave of absence from four-year qualification studies at universities in Seoul, Gyeonggi, Busan, and Daegu, where they live, based on the total population. It was carried out over 20 days from January 27 to February 15, 2021, and the sampling method was randomly selected at the convenience of the researcher. The sample size was calculated using the G*Power 3.1.9.4 program, and the minimum number of samples was determined to be 130, by applying a significance level of .05, power of .95, and an effect size of 15. However, while using a questionnaire, to secure the study’s validity, it is appropriate to generate a sample that is five to ten times larger than the total number of questions [15]; therefore, this study distributed 400 questionnaires. Finally, it utilized 331 data pieces and excluded 69 that had missing values ​(Fig. 1).

## Study tools

### Media exposure level

For the purpose of this study, media exposure levels were assessed by modifying a scale [32] related to news perception. Each question was measured using a 5-point Likert scale, with a total of five statements (1: not at all, 5: very much so). The statements posed were “When you talk to people around you, you can easily adhere to COVID-19 prevention rules,” “It is easy to access COVID-19 prevention rules on various SNS and messaging services such as Twitter, Facebook, Band, and Kakao Talk,” “If you browse the internet, you will be able to easily access the COVID-19 Prevention Rules,” “If you read Internet-based media or paper newspapers, you can easily look up the prevention rules for COVID-19,” and “The TV news makes it easy to learn how to prevent COVID-19.” Interpretation implies that a higher total score indicates a higher level of media exposure to COVID-19 prevention rules. The reliability of the study was as follows: Cronbach’s α = .77.

### Ego-gram

The index of the Ego-gram Check List was devised by John Dusay [33] and standardized and used by the Korea Transactional Analysis Association. It was utilized to determine the personality characteristics of selected participants in this study. The ego-gram contained five ego states (CP, NP, A, FC, and AC) and 50 questions (10 questions for each section). Ego-grams were measured using a 5-point Likert scale (0: not at all, 4: always yes), and for each question, the score ranged from 0 to a maximum of 40 points; thus, a higher score indicated a higher psychological energy level. The reliability of each ego state in this study was measured using Cronbach’s ɑ and results are as follows: CP: .76, NP: .80, A: .79, FC: .64, AC: .78.

### Distancing in daily life

Distancing in daily life (Guideline on May 6, 2020) includes personal and collective quarantine guidelines and refers to certain lifestyle and social structure improvements that have been developed to harmonize infection prevention and containment activities to prevent the spread of COVID-19 [6]. Particularly, personal quarantine comprises five key and four auxiliary rules that all citizens are urged to follow. This study focused on personal quarantine and selected eight suitable rules based on expert advice (two professors who were majoring in health management policy) and previous studies [34–37]. The rules were: “I wear a mask (on the premise that it is not mandatory),” “I stay home for 3 - 4 days if I’m sick,” “I maintain a distance of two arms’ length between other people and myself,” ‘I wash my hands for 30 seconds,” “I cough into my sleeve,” “I ventilate my personal spaces at least twice a day,” “I disinfect my personal spaces regularly,“ and “I maintain a healthy lifestyle“. These statements were measured on a 5-point Likert scale (1: Not at all, 5: Very much so). The participants’ scores ranged between 8 and 40 points; the higher end of the score was interpreted as “compliance with distancing in daily life.” The reliability of the study was as follows: Cronbach’s α = .70.

### Characteristics of the participants

Four items were used to represent the participants’ characteristics: sex, age, religious status, and monthly household income. Based on the literature, this study identified these variables [4, 6, 7, 34] as influential for social norms or attitudes. Specifically, in this study, these characteristics were as follows: sex (male [0], female [1]), age (18–20 years [0], 21–24 years [1], 25–30 years [2]), religious status (yes [0], no [1), monthly household income (KRW 1,990,000 or less [0], KRW 2,000,000-3,990,000 [1], KRW 4,000,000-5,990,000 [2], KRW 6,000,000-7,990,000 [3], KRW 8,000,000 or more [6]).

### Data collection

Data collection was conducted online from January 27 to February 15, 2021, for ‘participants who met the selection criteria of the study. The participants were recruited after the researchers wrote a recruitment notice in the online community of each university and sent an online survey form to be filled in by those who wished to participate voluntarily. A research statement containing research methods, procedures, and risks was presented to the participants, and only those who checked the “consent” box online to convey their approval could proceed with the survey.

### Statistical analysis

The SAS 9.4 program was used for the analysis of the data collected in this study. First, a frequency analysis, t-test, and one-way analysis of variance (ANOVA) were performed to understand the average difference between the demographic factors and distancing in the daily life of the participants. Second, we analyzed the mean and standard deviation of the major variables using descriptive statistics. Third, we conducted a hierarchical regression analysis presented in a previous study [38] to understand the moderating effect of ego-grams on the relationship between the media exposure level and distancing in daily life. To understand this moderating effect, the interaction variable, which is the product of the independent and moderator variables, must be utilized as the input. However, there was a threat of multicollinearity occurring in this case. Therefore, in this study, all variables except the dummy variables were mean-centering, and the analysis was conducted after confirming that there would be no problem regarding multicollinearity (variance inflation factor [VIF]: less than 10). The moderating effect model is represented by the following formula:

$$\mathrm Y=\beta_0+\beta_1X+\beta_2Z+\beta_3XZ+e$$

Here, X represents the independent variable, Z represents the moderator variable, and XZ represents the interaction term. If the interaction term β3 is significant, it is considered to have a moderating effect. If the moderating variable (β2) is significant, it is concluded that a quasi-moderator exists. Conversely, if the moderating variable (β2) is not significant, it is defined as having a pure moderator [39].

### Ethical consideration

Before this study commenced, the researchers received approval from the Institutional Review Board of Korea University for its purpose and method (Approval number: KUIRB-2021-0023-01). Prior to the survey, the first screen of the online survey provided a statement that included the study purpose, related details, procedures, benefits, participation risks, and participants’ right to withdraw at any time during the study. Furthermore, the participants were informed that the collected data would be used solely for research purposes, and that the researcher would utilize a locking device to protect participants’ personal information and discard this (personal data) after the completion of the study.

## Results

### The average difference between the participants’ general characteristics and distancing in daily life

Table 1 shows the average differences between the participants’ demographic characteristics and their distancing in daily life. First, based on sex, this study had a slightly higher proportion of female participants (143 men [43.2%] and 188 women [56.8%]). Regarding age, the various groups were distributed as follows: 21–24 (190 people [57.4%]), followed by 18–20 (81 people [24.5%]) and 25–30 (60 people [18.1%]). Regarding the presence or absence of religious beliefs (religious status), 107 people (32.3%) answered “yes,” and 224 people (67.7%) answered “no,” confirming that most of them did not have any religious beliefs. In terms of monthly household income, the “KRW 4,000,000-5,990,000″ group had the highest number of participants with 102 people (30.8%), followed by the “KRW 8,000,000 or more” group with 71 people (21.5%), the “KRW 2,000,000-3,990,000″ group with 66 people (19.9%), the “KRW 1,990,000 won or less” group with 51 people (15.4%), and the “KRW 6,000,000-7,990,000 won” group with 41 people (12.4%); this result confirmed that the income-related distribution was generally even. Finally, the average difference between the participants’ demographic characteristics and their distancing in daily life was confirmed. The results for sex (t = −.59, *p >* .05), age (*F* = 1.11, *p >* .05), religious status (t = −.29, *p >* .05), and monthly household income (F = .37, *p >* .05) were not significantly different.

**Table 1:** General characteristics of the participants and differences in distancing in daily life (*N* = 331)

Variables Category		N(%)	***t or f***	Post-hoc test
Sex	Men	143 (43.2)	−.59	–
	Women	188 (56.8)		
Age (Years)	18–20	81 (24.5)	1.11	–
	21–24	190 (57.4)		
	25–30	60 (18.1)		
Religious status	Yes	107 (32.3)	−.29	–
	No	224 (67.7)		
Household Income	Under 1,990,000 won	51 (15.4)	.37	–
	2,000,000-3,990,000 won	66 (19.9)		
	4,000,000-5,990,000 won	102 (30.8)		
	6,000,000-7,990,000 won	41 (12.4)		
	Over 8,000,000 won	71 (21.5)		
Total		331 (100)		

p^*^<.05, p^*^*<.01, p^*^**<.001

### Descriptive statistics for major variables

Table 2 presents the descriptive statistics for the major variables. First, considering that the media exposure level ranges from at least 5 to 25 points, the average of 20.00 points can be considered a very high number. Meanwhile, the standard deviation was 3.14 points, thus indicating that the deviation in terms of the media exposure level among the university students was not large, which confirmed that the media exposure level regarding COVID-19 precautions was uniformly high among university students. The level of distancing in daily life received an average score of 30.03 points, thereby indicating that university students tend to comply with distancing rules in daily life. A closer examination of the detailed factors shows that the average score for “I wear a mask” was 4.84 points, which was found to be much better than that for any other rules. Other points were 3.76 points for “I stay at home for 3-4 days if I’m sick,” 3.47 points for “I maintain a distance of two arms’ length between myself and other people,” 3.66 points for “I wash my hands for 30 seconds,” 4.25 points for “I cough into my sleeve,” and 3.68 points for “I ventilate my personal spaces at least twice a day.” It was 2.83 points for “I disinfect my personal spaces periodically,” which was not followed well compared to other rules, and 3.53 points for “I maintain a healthy lifestyle.”

**Table 2:** Descriptive analysis of major variables (*N* = 331)

	M	SD	Range
Media Exposure Level	20.00	3.14	5–25
Distancing in Daily Life	30.03	4.69	8–40
- I wear a mask	4.84	0.46	1–5
- I stay home for 3 ~ 4 days if I’m sick	3.76	1.00	1–5
- I maintain a distance of two arms’ length between myself and other people	3.47	1.01	1–5
- I wash my hands for 30 seconds	3.66	1.01	1–5
- I cough into my sleeve	4.25	0.96	1–5
- I ventilate my personal spaces at least twice a day	3.68	1.18	1–5
- I disinfect my personal spaces regularly	2.83	1.21	1–5
- I maintain a healthy lifestyle (regular exercise, balancing nutrition, etc.)	3.53	1.13	1–5
Ego-gram			
- CP (Critical Parent)	14.85	5.73	0–40
- NP (Nurturing Parent)	25.45	5.81	0–40
- A (Adult)	23.45	5.82	0–40
- FC (Free Child)	21.66	5.14	0–40
- AC (Adapted Child)	17.30	6.37	0–40

The ego-gram is divided into five categories, with an average score of 14.85 points for CP, 25.45 points for NP, 23.45 points for A, 21.66 points for FC, and 17.30 points for AC. Considering that each score ranged up to 40 points, it can be interpreted that the psychological energy level of university students was generally low. Furthermore, the standard of deviation was identified as ranging from at least 5.14 to 6.37 points, thus indicating that there was some ego deviation among university students.

### Types and characteristics of ego-gram

**Fig. 2:**
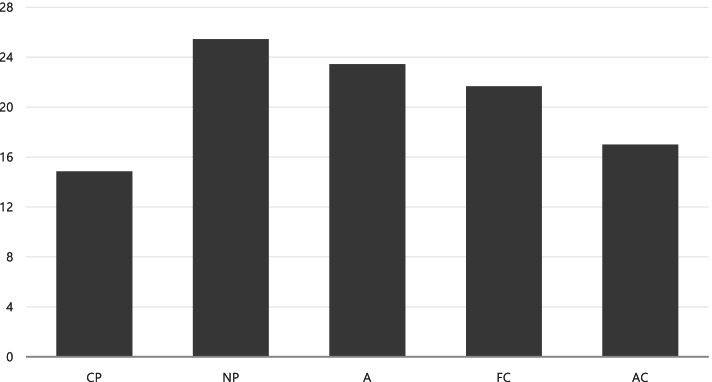
Ego-Gram Profiles of Participants

The ego-gram type of the university students who participated in this study showed a high NP and a low CP, A, FC, and AC round type (Fig. 2). The round type includes a few problems regarding interpersonal relationships, and it is a type that often appears among people who emphasize “humanity”—individuals who affirm themselves and others. The ego-gram can be classified into several types, along with seven representative types, including commitment, assertion, and conflict [20, 39]. The detailed types (patterns) of this study were representative of the round type (high NP), rational type (high A), and a commitment type (high NP and low FC).

### The moderating effect of the ego-gram on the relationship between media exposure level and distancing in daily life among university students

Table 3 presents the results of using university students’ distancing in daily life as a dependent variable, and inputting the demographic factors, media exposure level, ego-gram, and interaction variables between media exposure level and the ego-gram. First, Model I, which includes demographic factors and media exposure levels as variables, had an explanatory power of 5.3%, and an F value (F = 3.649, P < .01) that was significant. Sex (β = .036, *p >* .05), age (β = .045, *p >* .05), religious status (β = .017, *p >* .05), and monthly household income (β = .026, *p >* .05) were not significant, and only the media exposure level (β = .334, *p* < .001) was confirmed to be significant. In short, it could be said that higher levels of media exposure indicate better distancing in daily life.

**Table 3:** The Moderating effect analysis of the ego-gram on the relationship between media exposure level and distancing in daily life among university students (*N* = 331)

		Model I		Model II		Model III	
		B(S.E)	*β*	B(S.E)	*β*	B(S.E)	*β*
Control Variable	Sex	.339(.535)	.036	.321(.530)	.034	.440(.525)	.047
	Age (Years)	.322(.412)	.045	.387(.407)	.054	.509(.409)	.071
	Religious status	.168(.544)	.017	.508(.549)	.051	.421(.540)	.042
	Household Income	.091(.191)	.026	.064(.190)	.018	.103(.187)	.029
Independent Variable	Media Exposure Level(1)	.334(.081)	.224^***^	.301(.084)	.202^***^	.240(.087)	.161^**^
Moderator Variable	Ego-gram (CP)			.087(.053)	.106	.066(.053)	.080
	Ego-gram (NP)			.167(.050)	.206^**^	.164(.050)	.203^**^
	Ego-gram(A)			.030(.049)	.037	.032(.049)	.039
	Ego-gram (FC)			−.027(.052)	−.030	−.005(.053)	−.005
	Ego-gram (AC)			−.081(.049)	−.109	−.073(.048)	−.099
Interaction	(1) X (CP)					.030(.018)	.099
	(1) X (NP)					.004(.014)	.017
	(1)X(A)					.033(.014)	.138^*^
	(1) X (FC)					−.016(.016)	−.056
	(1) X (AC)					−.028(.016)	−.116
Model Fit	R^2^(Adj. R^2^)	.053(.039)		.091(.0063)		.139(.098)	
	R^2^ Change	.053		.038		.048	
	F	3.649^**^		3.220^**^		3.388^***^	

Sex: 0 = Men, Religious status: 0 = Yes

p^*^<.05, p^*^*<.01, p^*^**<.001

Model II, which had five ego-grams (moderator variables), had an explanatory power of 9.1%, and F values (*F* = 3.220, P < .01) were significant. CP (β = .106, *p >* .05), A (β = .037, *P >* .05), FC (β = −.030, *P >* .05), and AC (β = −.099, *p >* .05) were not significant, and only NP (β = .206, p < .01) was found to have significant effects. Therefore, a higher propensity for NP indicated better compliance with distancing in daily life.

**Fig. 3:**
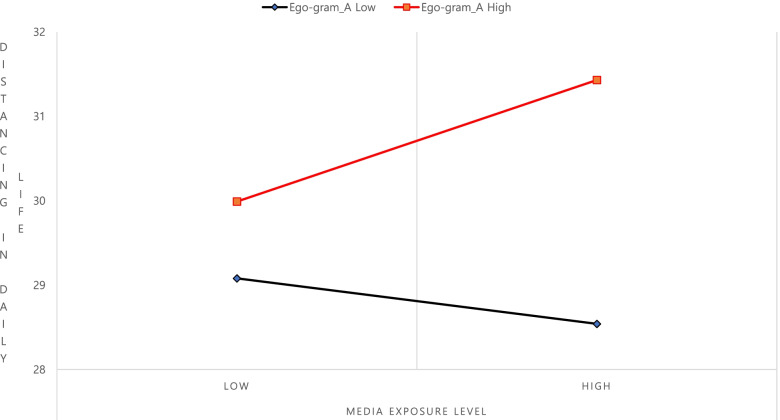
Interaction effects of media exposure and distancing in daily life on the ego-gram

Model III, which used an interaction variable that was multiplied by the media exposure level and the ego-gram, had an explanatory power of 13.9%, and an F value (*F* = 3.388, *P* < .001) that was significant. Previous studies suggest that social science studies, such as surveys, have an effect if the coefficient of determination is more than 13% [40]. The interaction variables for the media exposure level and A were statistically significant (β = .138, *p* < .05). Thus, the level of distancing in university students’ daily life was not entirely determined by their level of media exposure; the ego state did have a moderating effect. On the contrary, the moderating effects were also classified as pure and quasi-moderating effects, in which A (β = .039, P < .05) was shown to have a significant effect, indicating the existence of quasi-moderating effects. Figure 3 reveals that, among the ego states of university students, high A was greatly affected by the media exposure level, whereas for low A, the media exposure level had a relatively low influence. In short, a higher media exposure level and a higher ego state for A indicated greater compliance with an attitude towards practicing distancing in daily life.

## Discussion

This study investigated how the media exposure level to COVID-19 prevention rules could affect university students’ compliance with distancing in daily life rules and verified the moderating effect of ego-grams on this relationship. We thus examined the influence of distancing in daily life rules on individual life and explored the necessity of various quarantine-related programs while considering the specific characteristics and their policy implications. The analysis results and implications are as follows:

First, the analysis of university students’ recognition levels regarding the major variables selected showed that media exposure levels and distancing in daily life were above average; furthermore, the ego-gram types were round with high NP. A closer examination showed that the overall average for distancing in daily life was 30.03, which is somewhat high and suggested that university students properly complied with the distancing rules. Among the sub-factors, the rule “I wear a mask” had the highest adherence at the personal prevention level, with an average of 4.84 points, followed by “I cough into my sleeve,” with an average of 4.25 points. Subsequently, the rule “I disinfect my personal spaces regularly” and “I maintain a distance of two arms’ length between myself and other people” had a relatively poor adherence, with average scores of 2.83, and 3.47, respectively. Similar results have been observed in previous studies. The results of a survey on the conditions for wearing a mask among some local residents before mask-wearing was made compulsory [41] reported that the mask-wearing rate was 97.86%, thus indicating that most people wore masks. When another survey examined perceptions regarding the most important rule in distancing in daily life, “wearing a mask” ranked first, thereby showing the same outcome as our study results [42]. Wearing a mask has been shown to have the greatest effect in preventing COVID-19 infections, and it is reported to reduce the rate of transmission of COVID-19 [43]. The average score was 20.00 points, which indicated that the participating university students had experienced a high level of media exposure regarding COVID-19. This result can be confirmed through the findings of previous studies; reportedly, internet use rates are highest among people in their 20s compared to other age groups, and they tend to use Twitter, social networking services (SNS), news, and communication in various ways [44]. A study that targeted people in their 20s analyzed the level of exposure to media, including newspapers, broadcasting, and the internet, and found that the total scores were above average [45]. Furthermore, when examining the level of social media exposure by age group in the context of COVID-19, the college group was found to have higher exposure [46]. Media exposure levels were analyzed as an important factor in this study; we will address this issue in detail later. A subsequent examination of the ego-gram results showed that only NP, A, and FC were above the average value (20 points) and that the rest were below the average value; therefore, the psychological energy level of the participating university students could be interpreted as being below average. Regarding the ego type, the round type was observed, and as mentioned above, it was confirmed that there were a few problems in interpersonal relationships which showed a tendency to emphasize “humanity” [47]. These results can be understood by examining the current experiences of university students. In short, modern society demands that individuals maintain a mature personality in interpersonal relationships but must also be fiercely competitive. Therefore, university students experience situations in which they continuously undergo personality development through self-help groups and volunteer activities to grow into mature adults. These findings are supported by previous studies [48], which showed that voluntary activities acted as a stepping stone for promoting personal growth, community consciousness, and social responsibility among university students; other studies [22] demonstrated that voluntary activities themselves helped university students to form ego states. Overall, there was a difference in the increase or decrease in the university students’ levels of awareness based on ego gram factors. It is, therefore, necessary to adopt an integrated approach when examining them in detail and organizing quarantine rules.

Second, an analysis of the factors that affect distancing in daily life showed that sex, age, religious status, and monthly household income were not significant, while media exposure level and NP (one of the ego-grams) were significant. In particular, media exposure levels were identified as the most influential factor with regard to distancing in daily life. In short, a higher media exposure level among university students and their higher NP propensity indicated better compliance with distancing in daily life rules. This result suggests that media exposure level was the most influential factor in predicting university students’ distancing behavior in daily life. It is supported by a study on the elderly, which reported that a higher frequency of exposure to negative images through media (e.g., TV and SNS) indicated a higher chance of developing a negative attitude [20]. Another study examined the relationship between social media exposure level and political participation among university students with relatively low political interest and activeness compared to other age groups, and established that the media exposure level acted as an influential factor for political participation and increased participation behavior [49]. Additionally, media is an important influencing factor when promoting distancing, which is in line with the results of the study [31]. A study that explored the relationship between sociality and volunteer activity based on the ego status of university students volunteering showed that the factors affecting each ego status were different and that the type of NP had the greatest influence on volunteer activity participation [22]. It shows the need for policy support that considers media exposure levels to COVID-19 prevention rules and the propensity of NP to improve and manage distancing levels in the daily lives of university students. It specifically suggests that improving media exposure levels among university students could present an effective intervention direction.

Third, an examination of the moderating effect of ego-grams on the relationship between levels of media exposure to COVID-19 prevention rules and distancing in daily life among university students revealed that the propensity of A had a moderating effect. In short, when university students’ media exposure levels affect their distancing in daily life, a higher propensity toward A indicates a stronger attitude of complying with distancing in daily life. The type A indicates the propensity to judge and objectively view things based on facts and the ability to make decisions based on various types of information [50]. In short, it can be said that a higher propensity toward A indicates a stronger likelihood of abiding by social norms such as distancing in daily life. These results are supported by a previous study [15] that reported that among ego-gram propensities, A had a moderating effect on the relationship between depression and stress among university students based on their smoking status. However, CP, NP, FC, and AC had no moderating effect on this propensity; the tendency to have positive (+), positive (+), negative (−), and negative (−) effects was confirmed. Reportedly, regarding AC, a more positive (+) tendency indicated greater compromise and cooperation, which differs from the findings of this study. It can be inferred from this study’s population distribution in which most participants accounted for 81.9% of the relatively low age group (18–24 years). Furthermore, internal stress from sudden changes in daily life patterns, including a decrease in external activities and social gatherings due to COVID-19, may have acted as a contributing factor. Future studies are necessary to closely examine these points.

Finally, additional analysis was performed using the data of representative ego-types (round, rational, and commitment) found among university students, which this study had identified. First, because of the confirmation of the factors that affect the distancing in daily life rules, the media exposure level (β = .840, P < .001) had a significant effect on the data of the rational type but was not significant for the remaining data. In short, the influence of media exposure levels on COVID-19 implied that, among the university students, those with rational egos were more affected. Thereafter, this study examined the moderating effect of the ego-gram on the relationship between the media exposure level and distancing in daily life and confirmed that there was no moderating effect in any of the data.

Based on the discussion above, there are a few policy implications for managing and promoting the level of distancing in daily life among university students. First, it is necessary to continuously monitor media exposure levels, which have been identified as the most influential factor for the level of distancing in daily life among university students; it is also important to increase the frequency of such exposure. Divided into 1–2 phases, the 1st phase surveys the degree of media exposure regarding COVID-19 in universities (i.e., the place where university students spend most of their time) by using various methods such as online questionnaires. The 2nd phase aims to promote, maintain, and manage the level of media exposure through education in connection with community resources (health centers, NGOs, etc.) among university students with low media exposure levels to COVID-19 prevention rules. Moreover, the frequency of such media exposure should also increase. Currently, public institutions and various related institutions operate several channels (Facebook, Instagram, YouTube) to spread information, which should be actively utilized. In short, it is necessary to expand existing channels, including TV news, Internet media, and newspapers, to promote COVID-19 prevention rules in various ways in conjunction with social media such as Facebook, Instagram, and YouTube, which are mostly used by university students.

Second, it is necessary to explore various quarantine-related programs that consider the characteristics of the ego. At present, it is difficult to organize customized quarantine programs that consider the characteristics of each individual. Therefore, it is necessary to organize and expand activities and programs that involve campaigns, mentoring, ambassadorships, and education about COVID-19 by utilizing the egos of NP and A, which have been identified as indicating relatively strong compliance with social norms. University students tend to judge their actions and decisions and compare them to their peer groups as they develop from immature young adults into mature adults. If we make appropriate use of their tendencies, they can have a positive impact not only on university students but also on members of society. Additionally, it is necessary to focus on the ego of A, which has a moderating effect. In this study, the ego-A of university students was low (at about 23 points), whereas in studies involving mature adult participants (e.g., 119 paramedics and early childhood teachers) and the same scale, ego-A was higher than the average value (about 32 points) [51, 52]. It is presumed that this difference can be mainly attributed to A’s propensity to appear mainly in mature adults who view reality objectively and make decisions based on systematic and rational judgments. Therefore, this finding suggests that raising the propensity toward A appropriately (i.e., to the average value found among adults) in the university student group, which has a relatively low propensity toward A, can be an important intervention direction for norm compliance. Previous studies have supported this fact, and reports suggest that a higher ego of A indicates a higher level of rationality and that such qualities of a democratic citizen who abides by law and order can have a positive effect on community life [53]. Reportedly, the propensity toward A does not simply increase with age and experience; rather, it differs depending on the amount of information gained through education [27]. Thus, in the long term, an approach to systematically build and expand a program that can check, manage, and promote A propensity and ego status at the university and community levels may be required. In other words, considering the situation in which new infectious diseases such as COVID-19 occur every 4–5 years [54], these efforts are to prepare for future pandemics and to lay the foundation to improve the level of individual quarantine prevention measures.

## Conclusions

This study investigated the moderating effect of the ego-gram, an indicator of individual personality traits, on the relationship between media exposure levels to COVID-19 prevention measures and distancing in daily life among university students aged 18–30 years. The results showed that the level of media exposure was the most influential factor for distancing in daily life among university students; simultaneously, it acted as a major predictor of distancing in daily life levels. In short, it is necessary to continuously monitor media exposure levels among university students and increase their frequency of exposure. Among other factors that significantly influence distancing in daily life, NP was found among the ego states, and A was confirmed to have a moderating effect. This result suggests that various COVID-19-related quarantine programs should be organized and expanded by using ego groups that have been confirmed to adhere to the norms properly.

Finally, this study had some limitations, based on which we suggest follow-up research. First, the results of this study cannot be generalized because of the limitations of the study participants; that is, only university students aged 18–30 years were examined, and a relatively small number of cases (331 people) were used. In the future, for generalization purposes, a study considering national representativeness, such as various age groups and a larger number of people, should be conducted. Second, it was not possible to comprehensively analyze the various factors that influence distancing in daily life. In particular, regional factors were used to a limited extent. It is desirable to measure factors by allocating all regions equally, but in this study, the four cities with the largest number of inhabitants were selected based on the total population. In the future, it will be possible to measure the effect more accurately by examining a wide range of factors such as occupation, psychological factors, and health behavior, in addition to these aspects. Third, the measurement method was limited. In this study, variables were measured in a self-reported manner, and they may overstate the actual behavior level. Fourth, this study included cross-sectional studies that present difficulties in identifying causality over time. In other words, in a situation where COVID-19 continues for a long duration, it is predicted that individual behavioral change factors will vary with time. Therefore, to confirm the relationship between media exposure level and distancing in daily life, it is necessary to conduct a longitudinal study. Fifth, there are few investigations on the effect of ego intervention on distancing in daily life, and the effect was only partially verified in this study. In the future, the results of this study can be supported by further research.

## Data Availability

The datasets used and/or analyzed during the current study are available from the corresponding author upon reasonable request.
